# Molecular dynamics study of strengthening mechanism of nanolaminated graphene/Cu composites under compression

**DOI:** 10.1038/s41598-018-21390-1

**Published:** 2018-02-15

**Authors:** Shayuan Weng, Huiming Ning, Tao Fu, Ning Hu, Yinbo Zhao, Cheng Huang, Xianghe Peng

**Affiliations:** 1 College of Aerospace Engineering, Chongqing University, Chongqing, 400044 P. R. China; 2 Key Laboratory of Optoelectronic Technology & Systems, Education Ministry of China, Chongqing University, Chongqing, 400044 P.R. China

## Abstract

Molecular dynamics simulations of nanolaminated graphene/Cu (NGCu) and pure Cu under compression are conducted to investigate the underlying strengthening mechanism of graphene and the effect of lamella thickness. It is found that the stress-strain curves of NGCu undergo 3 regimes i.e. the elastic regime I, plastic strengthening regime II and plastic flow regime III. Incorporating graphene monolayer is proved to simultaneously contribute to the strength and ductility of the composites and the lamella thickness has a great effect on the mechanical properties of NGCu composites. Different strengthening mechanisms play main role in different regimes, the transition of mechanisms is found to be related to the deformation behavior. Graphene affected zone is developed and integrated with rule of mixtures and confined layer slip model to describe the elastic properties of NGCu and the strengthening effect of the incorporated graphene.

## Introduction

Graphene, as one of the most promising two dimensional material, has shown extraordinary intrinsic electrical, thermal and mechanical properties^1^. However, It is still quite difficult to directly apply graphene as structure materials due to its unique geometric features and the interfacial instability^2^. Using graphene as constituent phase in composites thus has been considered as a versatile method to make use of the excellent performance of graphene. Effective enhancement in strength and toughness was reported for various graphene-filling composites such as polymer^3^, ceramic^4^ and metal matrix composites^5,6^.

Graphene/metal composites inspire broad interests in investigating the structure and properties of graphene/metal interface because they are a key to the development of catalysis, sensors, hydrogen storage, and nanoelectronic devices^7^. Furthermore, incorporating graphene is proved to improve not only the designed functions but also the mechanical performances of the metal matrix phases, showing great prospects in engineering applications^8–11^. Researchers had successfully fabricated graphene/Cu, graphene/Al composites with uniform dispersion of graphene in metal powders and found various degrees of reinforcement in elastic modulus, hardness and tensile strength by no more than 1.0 wt.% graphene addition^12–15^. In 2013, nanolaminated graphene/metal composites reinforced by single layer of graphene were synthesized by Kim *et al*. The nanopillar compression test results showed ultra-high strengths of 1.5 GPa and 4.0 GPa for graphene/Cu and graphene/Ni nanolayered composites respectively^16^. Uniaxial compression tests were also carried out on graphene/Al nanolaminated micro-pillars^17^. It was found that the strengthening effects were related to the graphene concentrations and laminate orientation. 137% higher flow stress than pure Al pillars and crack deflection mechanism were observed. In addition, molecular dynamics (MD) simulations were also conducted and revealed that the incorporating graphene can improve the radiation damage resistance^18^, shock strength^19–21^ and ductility of nanolaminated graphene/metal composites^22,23^.

The load-bearing capacity and blocking dislocation propagation effect of graphene are believed to account for the reinforcement in graphene/metal composites^24–26^. Similar mechanisms have been demonstrated in nanolaminated metal composites, in which interface plays an important role in mediating deformation mechanisms and mechanical properties^27^. In nanolaminated metal composites, three kinds of strengthening mechanisms were proposed to describe the strength variation related to the lamella thickness: the Hall-Patch relationship, the confined layer slip (CLS) model, and the interfacial barrier strength mechanism^28,29^. nanolaminated metal composites with a critical layer lamella thickness were found to obtain optimal hardness or strength^30–32^. However, for nanolaminated graphene/metal composites, the underlying mechanisms, including the role of graphene/metal interface need to be further explored, especially at the atomic level. Besides whether these models used in nanolaminated metal composites can be applied to graphene/metal composites and the effect of lamella thickness on the properties of nanolaminated graphene/metal composites remains unknown.

Nanolaminated materials such as multilayered coatings often undergo out-of plane compression loading in actual working condition. Therefore, in this study, we carried out-of plane compression simulations on nanolaminated graphene/Cu (NGCu) composites by MD method to investigate the effects of incorporated graphene and the deformation mechanism. Effects of lamella thickness are also investigated by comparing mechanical performance of models with various lamella thicknesses.

## Simulation Details

### Interatomic potential

The embedded atom method (EAM) potential^33,34^ is proven to accurately depict the many-body atomic interactions in metallic systems and widely used to simulate the deformation behavior under various loading conditions^35,36^. Therefore the EAM potential is employed to describe the interaction of copper atoms. Reactive empirical bond order potential is used to depict the interaction between carbon atoms. While the interaction between carbon and copper atoms is described by Lennard-Jones (LJ) potentials which have been proven to successfully investigate the peeling^37^, thermal conductance^38^ and shear deformation^2^ of nanolaminated graphene/metal composites. The LJ parameters with equilibrium separation *σ*_(C-Cu)_ = 3.0825 Å, potential depth *ɛ*_(C-Cu)_ = 0.02578 eV and the cutoff *r*_c_ = 2.5*σ*_(C-Cu)_ are adopted^10,20,39^. These LJ coefficients have been successfully employed to simulate the interface cracking, radiation damage and high-speed impact processes of graphene (graphite)-copper systems^10,20,40,41^.

### Molecular dynamics model

**Figure 1:**
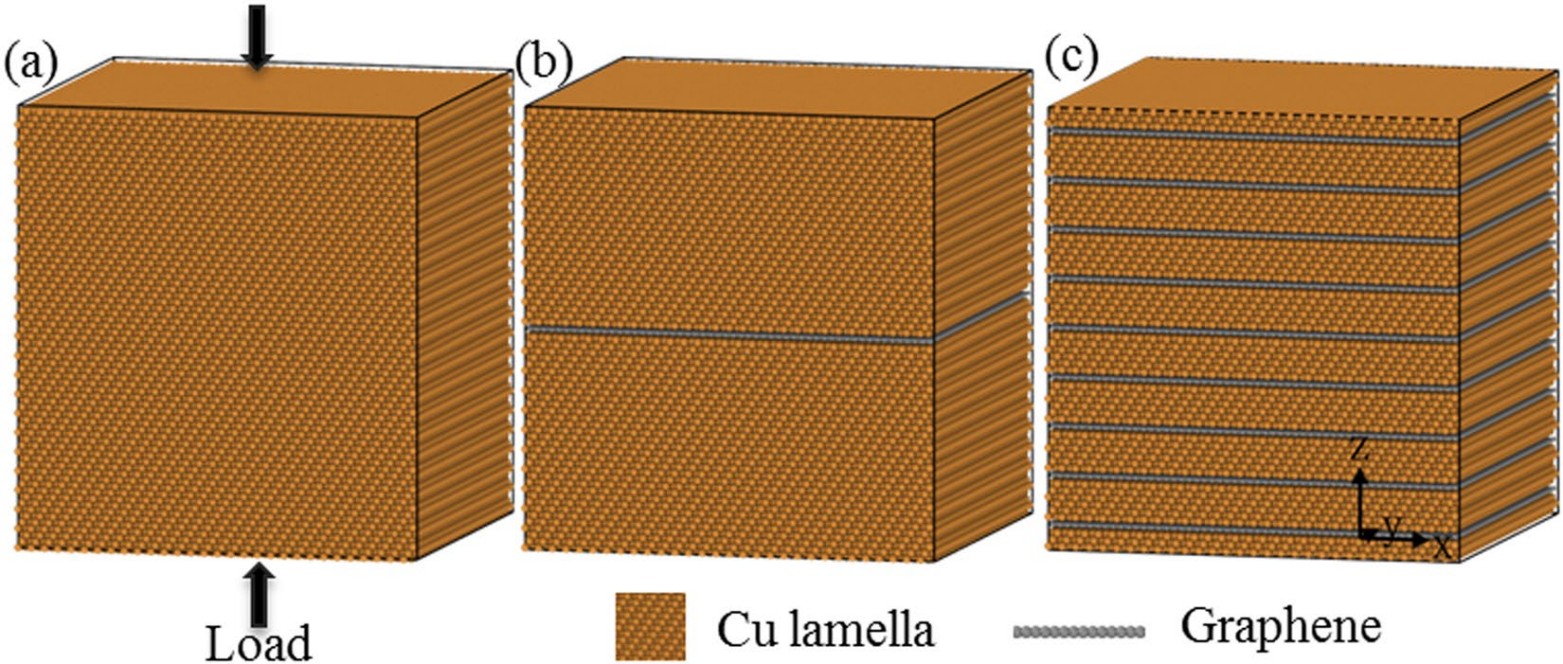
Schematics of (**a**) pure Cu, (**b**) NGCu_1_, (**c**) NGCu_7_ models.

There are wide reports about graphene growing on Cu (100) and (111) single crystals thin films. And the Cu (111) surface is found to grow higher quality monolayer graphene with high area coverage and short growth time^42^. Therefore, in the present work, we consider graphene packed on Cu (111) surface with the zigzag and armchair directions parallel to *x* and *y* directions respectively (Fig. 1). All the models are aligned with coordinate system defined by the Cu matrix crystallographic orientations as *x*/[11 $\bar{2}$], *y*/[$\bar{1}$ 10], *z*/[111]. The lattice mismatch between Cu and graphene lattice is around 3.5%^43^. In order to minimize the lattice distortion, the dimension of the MD models is selected according to Table 1, where *a*_cu_ = 3.615 Å, *a*_gra_ = 2.46 Å, *b*_gra_ = 4.26 Å. NGCu_*i*_ models are constructed with different interlayer distance between graphene layers characterized by the individual Cu lamella thickness *λ*_*i*_ = N_*i*_ [111]/3*a*_Cu_, N_*i*_ = 66, 32, 21, 15, 12, 9, 6 for *i* = 1–7. In addition, a pure Cu model is also constructed for comparison. The schematic of pure Cu and two representative NGCu models are illustrated in Fig. 1. NGCu_1_ has the biggest Cu lamella thickness 137.7 Å, while NGCu_7_ possesses the thinnest Cu lamella with *λ*_7_ equals to 12.5 Å. The total length of all the models in *x, y, z* direction is around 133, 127, 142 Å respectively.

**Table 1:** Crystal orientations and sizes of the models.

Model	Constituent	lx	ly	lz
NGCu_*i*_	Cu layer	30[11 $\bar{2}$]/2*a*_Cu_	50[$\bar{1}$ 10]/2*a*_Cu_	N_*i*_ [111]/3*a*_Cu_
	Graphene	54*a*_gra_	30*b*_gra_	Single layer

Before compression loading, the models are first optimized using the conjugate gradient algorithm to perform an energy minimization of the system, by iteratively adjusting atom coordinates. Then, the structures are further relaxed using a Nose-Hoover thermostat^44^ and a Nose/Hoover pressure barostat^45^. The system is kept at a constant temperature 10 K and the pressures in *x, y* and *z* directions are set to zero for the duration of 60 ps. Compression along *z* axis is subsequently applied to the models at a strain rate of 1 × 10^9^ s^−1^. During the compression process, the NPT ensemble is employed with Nose/Hoover barostatting to keep the pressure to be zero in x, y directions. Periodic boundary conditions are applied in three directions of all the models.

All MD simulations are conducted using the Large-scale Atomic/Molecular Massively Parallel Simulator (LAMMPS)^46^. And the Open Visualization Tool (OVITO) developed by Stukowski^47^ is employed for post-processing atomistic data obtained from MD simulation. The dislocation extraction algorithm (DXA)^48^ is used to identify the local environment of particles and assign a structure type (FCC, BCC, HCP, etc.) to each particle. This method can be further used to identify dislocations in a crystal and determine their Burgers vectors.

### Data Availability

The datasets generated during and/or analysed during the current study are available from the corresponding author on reasonable request.

## Results

### Stress-strain curves of models with various lamella thickness

**Figure 2:**
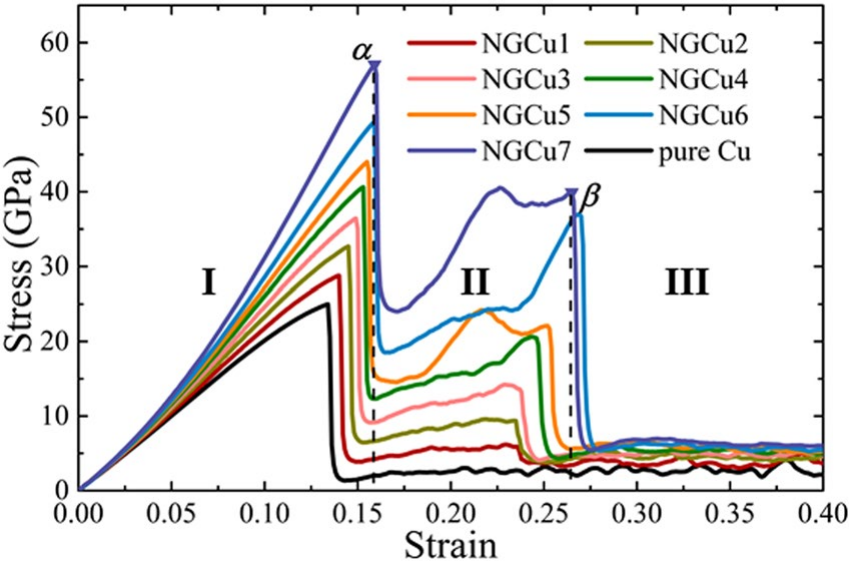
Compression stress-strain curves of pure Cu and NGCu models with different lamella thickness.

Figure 2 displays the obtained stress-strain (σ-ε) curves of pure Cu and 7 NGCu models under compression. It can be seen that the stress rises linearly with the strain up to the first peak stress in pure Cu model then goes to a steady flow state directly. While in NGCu models, two peak points labeled by *α* and *β* can be observed in each curve. The curves show nonlinear regimes before reaching the first peak *α*. Then stress drops sharply and gradually grows back to the second peak *β* before falling to the final plastic flow state. In this case, as shown in Fig. 2, the σ-ε curves of NGCu can be separated to 3 regimes: the elastic regime I, plastic strengthening regime II and plastic flow regime III. In elastic regime I, the Young’s modulus and yield stress of NGCu increases with the decreasing of *λ* and are all higher than that of pure Cu model. In plastic strengthening regime II, A strengthening effect caused by graphene reinforcement can be clearly observed in NGCu while it is absent for Cu model. The graphene exhibits more apparent strengthening effect with the smaller *λ*, resulting in a higher stress in of NGCu in regime II. In plastic flow regime III, the stress goes to a plastic flow plateau. And with the decrease of *λ*, the average flow stress rises. Notice that the obtained strength of pure Cu in the simulation is much higher than the experimental value which is usually no more than 2 GPa^49^. This difference should be ascribed to following reasons: (1) Time-step used in MD simulations is usually a few femtoseconds, leading to materials withstanding much higher strain rate than that in the actual experiments and achieve higher strength to some extent. Meanwhile behaviors such as diffusion process which often occurs with large time scales are ignored in simulations. (2) MD models possess ideal microstructure without pre-exsisting defects^30^, while experimental samples inevitably contain a variety of interstitials, vacancies, dislocations or grain boundaries, which affect the mechanical properties greatly. (3) The temperature used in the present simulations (10 K) is much lower than that in experiment.

### Deformation mechanism transition during deformation stage I-III

**Figure 3:**
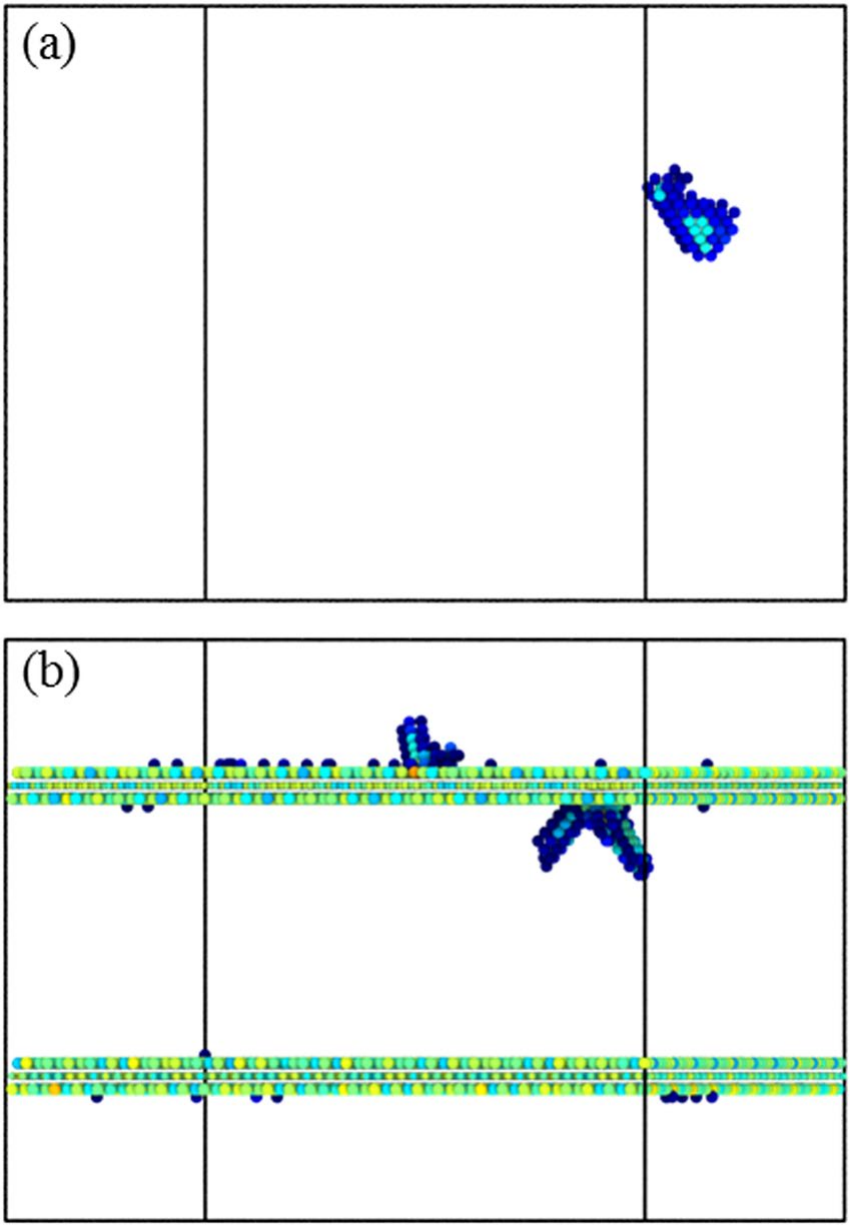
Atomic configurations colored by CSP of (**a**) pure Cu, (**b**) NGCu_2_ at the point *α*.

Figure 3 shows the atomic configurations of pure Cu and NGCu_2_ at the first peak point α, where the atoms in perfect FCC lattice have been removed, and the rest atoms are colored by their centro-symmetry parameter (CSP) for clarity. It can be seen that dislocation nucleates at point *α* in both models. Shockley partial dislocations nucleate homogeneously in pure Cu under compression, while dislocations emit from the graphene/Cu interface which in this way acts as a dislocation source. According to Fig. 2, the stress for dislocation nucleation in NGCu models is much higher than that in pure Cu, which seems to contradict the dislocation source role of the interface. It is commonly accepted that the critical shear stress τ_c_ is the important factor for dislocation nucleation at/near grain boundaries^50^. According to the work of Armstrong *et al*.^51^, τ_c_ has athermal and thermal component. And the thermal component is inversely proportional to the activation volume V_c_^*^of thermally activated process by taking τ_c_V_c_^*^ = 3.1 × 10^−20^ J. V_c_^*^ for graphene/Cu composites at yielding point is only one third of that in the pure Cu through experimental measurement^24^. Due to the distinct thermal activation behavior of graphene/Cu interface, a higher critical shear stress is needed for graphene/Cu composites to overcome in terms of dislocation nucleation, resulting in higher stress at point *α* of NGCu models compared with that in pure Cu in Fig. 2. This is consistent with the observation in compressive experiment by Armstrong *et al*.^51^.

**Figure 4:**
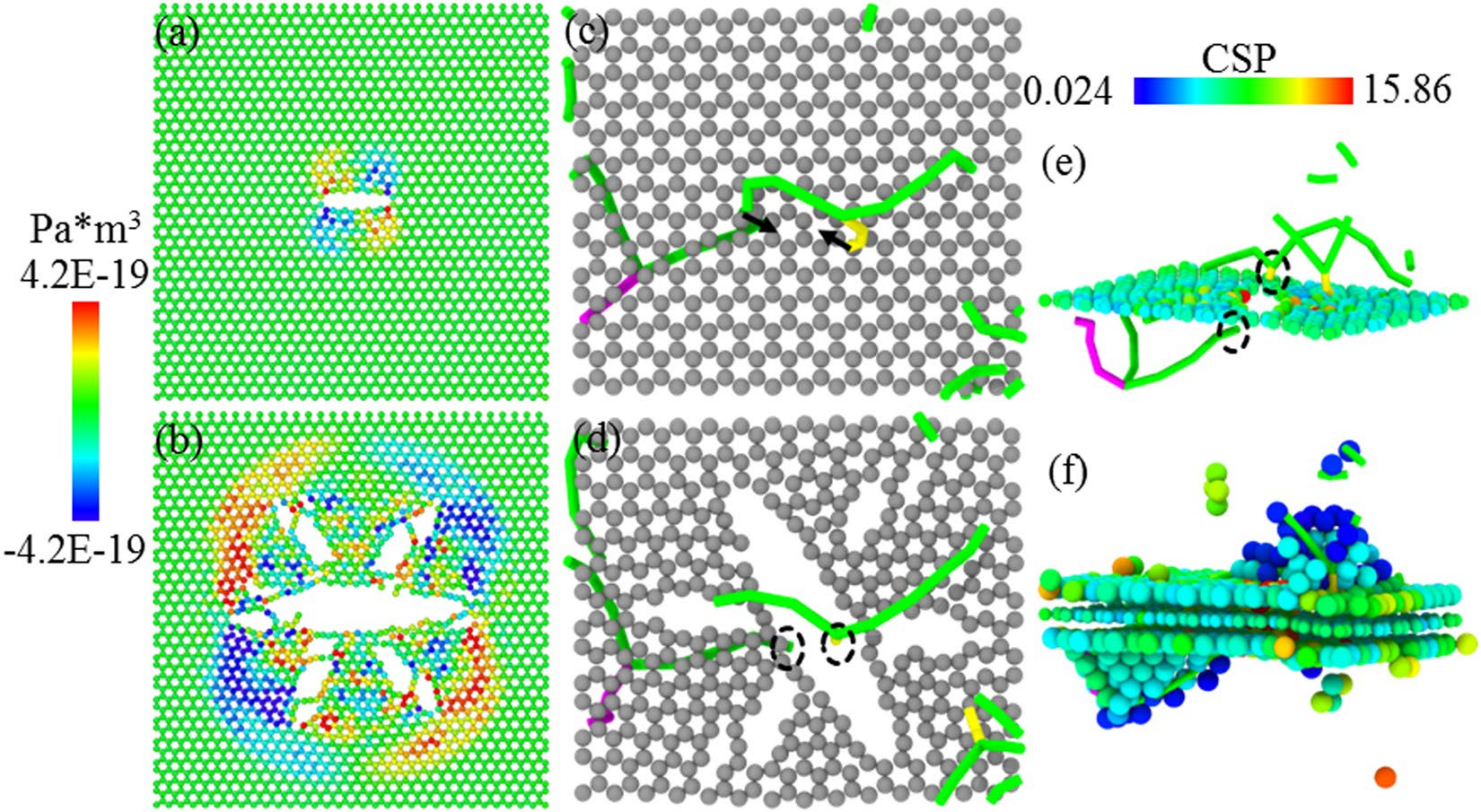
(**a**–**b**) The crack propagation of graphene in NGCu_2_ at the second peak point colored by σ_xy_, (**c**–**f**) crack formation in NGCu_1_: (**c**–**d**) the top view with only graphene atoms, (**e**–**f**) defect structures colored by CSP distributions.

Figure 4 shows the atomic configurations of graphene layers in NGCu_1_ and NGCu_2_ at the second peak point *β*. It is the fracture of graphene that leads to the sharp drop of the stress at point *β*. The C-C bonds break along x axis forming an initial crack with a length of 18 Å in NGCu_2_ leading to a symmetrically distributed shear stress σ_xy_ (Fig. 4(a)), which subsequently brings about 4 fractal crack growing along the other two equivalent zigzag directions in graphene (Fig. 4(b)). Similar process can be found in other NGCu models including NGCu_1_ shown in Fig. 4(d). Figure 4(c–f) illustrate the formation process of the initial crack. There are two Shockley partial dislocation lines lying above and beneath the graphene layer. The two dislocations move in two (111) plane inclined with the graphene respectively and gather together until being located at two sides of an array of graphene atoms along zigzag direction, along which an initial crack splits.

**Figure 5:**
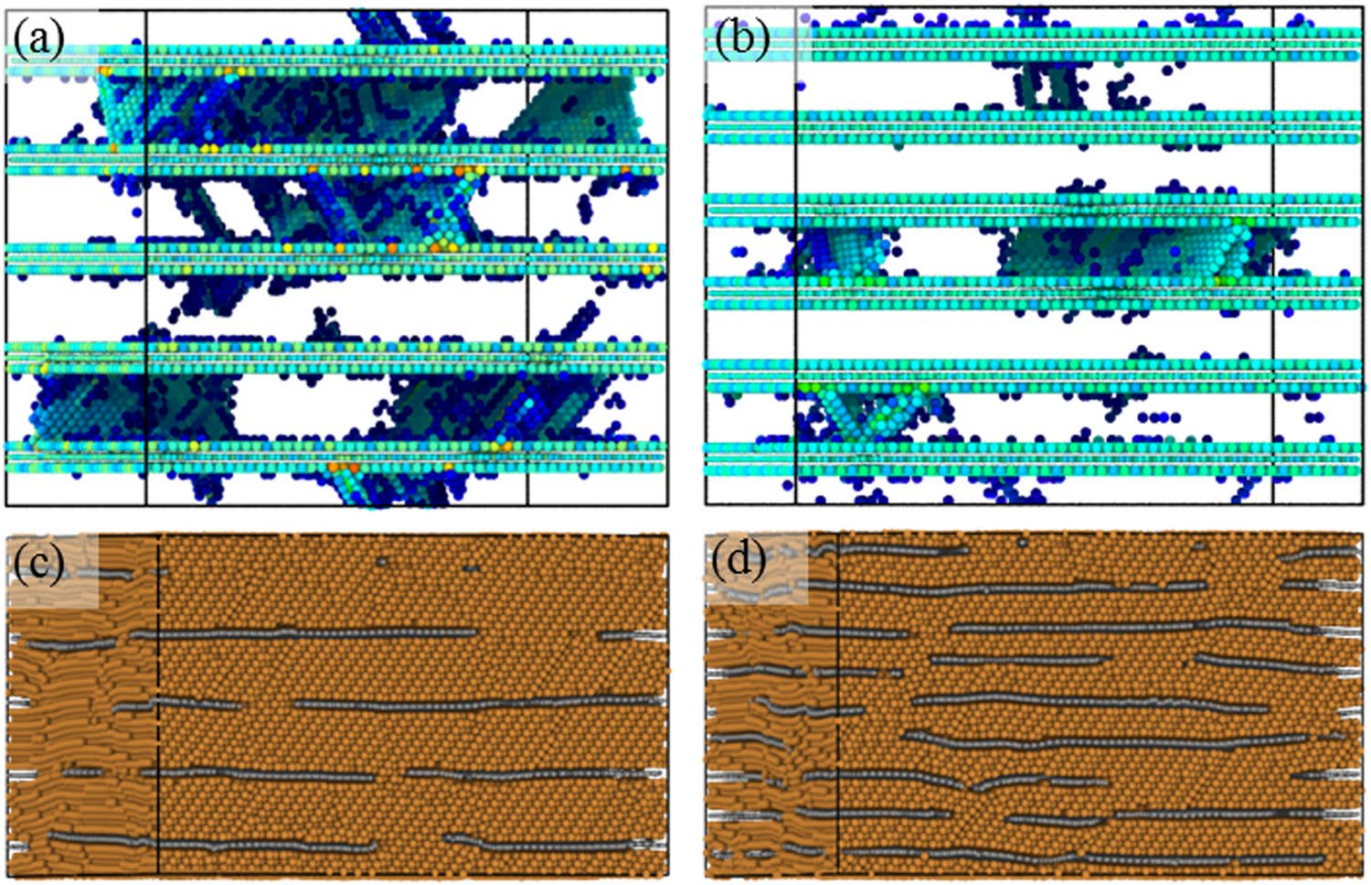
Atomic configurations of (**a**) NGCu_5_ and (**b**) NGCu_6_ in regime II colored by CSP, (**c**) NGCu_6_ and (**d**) NGCu_7_ in regime III colored by atomic type.

After dislocation nucleation, the compressive stress in stress-strain curves falls to a valley. The nucleation of dislocation is very difficult at small length scales which means that dislocations are strongly pined by the interfaces and need larger stress to emit^32,36^. Therefore, the stress increases with further loading in the plastic strengthening regime II of NGCu models as shown in Fig. 2. Figure 5(a,b) show the atomic configurations in regime II with only the defect atoms left. Dislocations propagate and slide until being obstructed by the graphene layers. Due to the high strength of graphene sheets, dislocation can hardly cut across the graphene to slide in adjacent layer. Therefore dislocations are confined in the Cu layer. Graphene layers are still capable to bear the increasing loading strain without any bond breaking, which refer to the rising stress of NGCu models in regime II. When the strain exceeds the value of the second peak *β*, the cracks initiate and propagate in all graphene layers and the graphene layers are teared into fragments (Fig. 5(c,d)), the stress thus goes to a plastic flow plateau in plastic flow regime III from then on (Fig. 2).

## Discussion

### Variation of elastic properties of NGCu with decreasing lamella thickness

**Figure 6:**
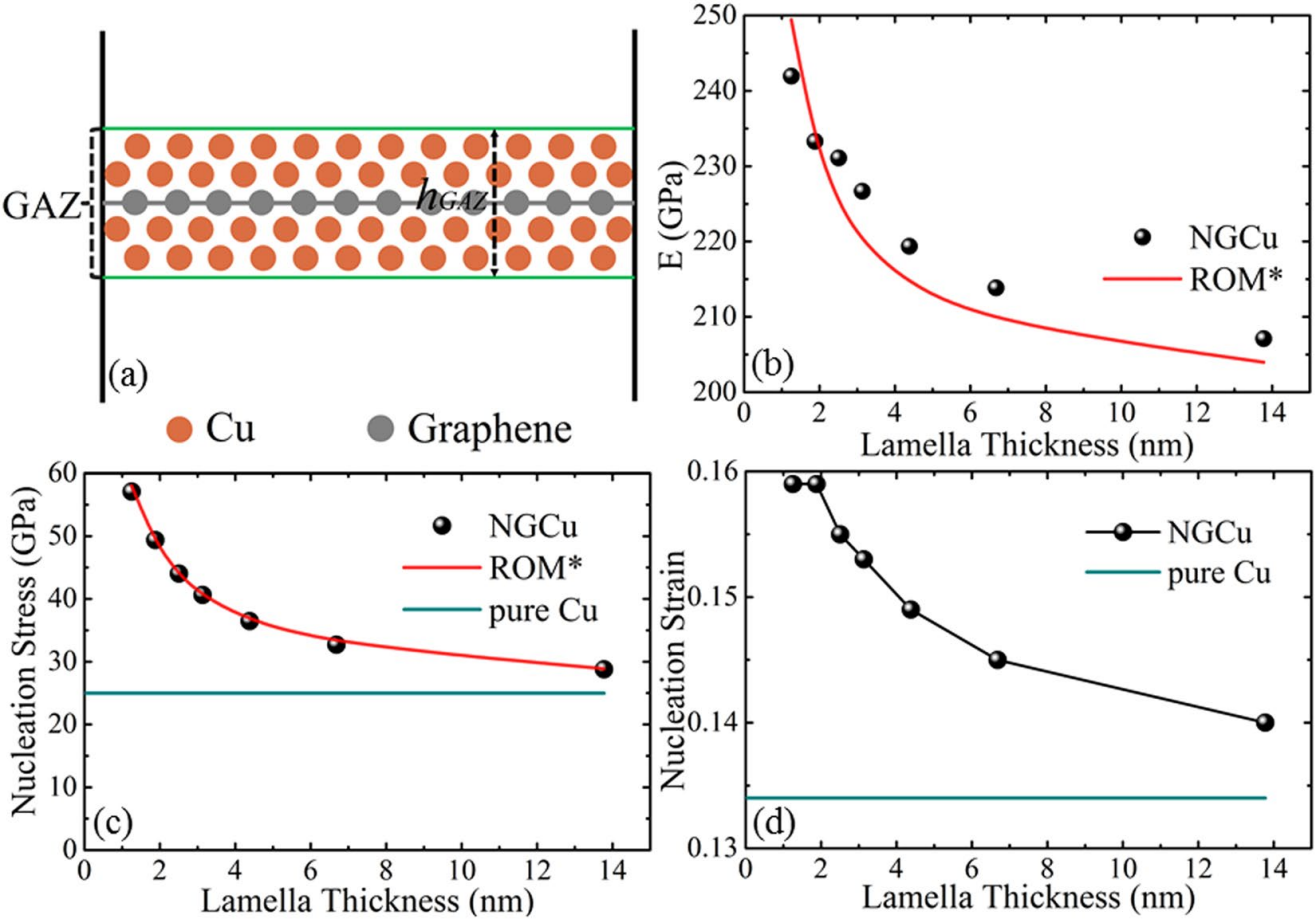
(**a**) The schematic of the graphene affected zone (GAZ), (**b**–**d**) variations of E, dislocation nucleation stress and strain with various Cu lamella thicknesses.

From Fig. 2, we can see that the NGCu models show weak nonlinearity in the elastic regime I with compressive strain progressing. And as the Cu lamella thickness λ decreases, the nonlinear elasticity becomes increasingly apparent depicted by a bigger and increasing tangent slope. The nonlinear elasticity should be ascribed to the instinct nonlinear elasticity of graphene monolayer with a form of *σ* = *Eɛ* + *Dɛ*^2 ^^52–54^. It is reported that the value of *D* is typically negative, so the second-order term is negative, the presence of which leads to a lessening of stiffness at high tensile strains and an increasingly stiff response at high compressive strains^52^. Noticing that in this paper the graphene is subjected to an out-of-plane compression which is different from the usual in-plane loading state in these references, the stiffness none the less increases with continued compressive strain. Young’s modulus *E* of each model is extracted from the obtained stress-strain curves in initial linear strain range of 0.0~0.040 and plotted in Fig. 6(b). The rule of mixtures (ROM) is widely used to describe the Young’s modulus, yield stress and the shear modulus of composites^2,32,55^. However, when imposing the ROM to fit the obtained *E* of different model using $\frac{1}{{E}_{{\rm{N}}{\rm{G}}{\rm{C}}{\rm{u}}}}=\frac{{V}_{{\rm{C}}{\rm{u}}}}{{E}_{{\rm{C}}{\rm{u}}}}+\frac{{V}_{graphene}}{{E}_{graphene}}$, the obtained *E*_graphene_ returned a negative value which means that the ROM cannot be applied to NGCu models using the equation mentioned above. It is known that the equation of ROM is obtain by considering the *ɛ*_NGCu_ = *ɛ*_Cu_ + *ɛ*_graphene_. While due to the unique geometric features of graphene (two dimensional), it is very tricky to impose compression test on graphene and quite challenging to obtain the compressive strain of graphene monolayer. So the Young’s modulus of graphene under compression is rarely obtained in both experiment and simulation. In addition, this equation of ROM treat graphene and Cu matrix in a simplified manner neglecting the interaction between the two phases.

Inspired by the concept of the approaches proposed by Mayeur *et al*.^56^, Cu in the region adjacent to the graphene is largely affected by the state of graphene at this scale, but the effect reduces as a function of distance. The deformation and stress state of these Cu atoms are constrained by graphene monolayer, so the mechanical properties of the Cu in region adjacent to the graphene should not be treated in the same way as the Cu far away from the graphene layer. In this case, a model in which a region extending from both sides of the graphene is defined as a graphene-affected zone (GAZ), is developed as illustrated in Fig. 6(a). Within this region, the mechanical response and properties of the Cu phases are predominantly controlled by the incorporated graphene. So the GAZ including the graphene monolayer and adjacent Cu as a bulk portion of the composites is employed to recalculate the mechanical properties. The equation of ROM is revised as: $\frac{1}{{E}_{{\rm{N}}{\rm{G}}{\rm{C}}{\rm{u}}}}=\frac{{V}_{GAZ}}{{E}_{GAZ}}+\frac{{V}_{Cu\ast }}{{E}_{Cu}}$, where *V*_Cu_* represent the volume of graphene unaffected zone excluded from the GAZ. The thickness *h*_*GAZ*_ is decided to include the second nearest layer of Cu atoms, beyond which the interaction between graphene and Cu are comparatively small and are neglected in our model. Thus *V*_GAZ_ and *V*_Cu*_ can be calculated respectively according to the thickness *h*_*GAZ*_. The value of *E*_*GAZ*_ is first calculated by solving the revised rule of mixtures with the parameters of model NGCu_7_. By substituting the Young’s modulus of pure copper *E*_Cu_ and the model *E*_NGCu7_, the corresponding volume fractions of affected zone *V*_GAZ_ into the equation of revised ROM, *E*_*GAZ*_ can be obtained. Then the obtained *E*_*GAZ*_ is used to recalculate *E*_NGCu_ of all other models. The above revised ROM is labeled as ROM* in the present work. Figure 6(b) indicates that the GAZ integrated ROM* fits well with the obtained *E* of models with decreasing Cu lamella thickness λ.

Figure 6(c,d) show the stress and strain of the first peak point α respectively. As discussed above, the dislocation nucleates at this point. It can be found from Fig. 6(c,d) that incorporating graphene can delay the nucleation process by a larger stress and strain. There is no dislocation reaction happening before point α, so the delayed dislocation nucleation should be attributed to the loading-bearing effect of the incorporated graphene. As ROM can be applied to describe the yield stress^32,57^, we used the ROM* taking the GAZ into account to fit the dislocation nucleation stress in Fig. 6(c). The curve being described by the ROM* is in good agreement with those MD results. As to the dislocation nucleation strain, with the Cu lamella thickness *λ* decreasing from 13.8 nm to 1.9 nm, the strain gradually increases. While, the nucleation strain goes to a plateau with λ beneath 1.9 nm, which indicates the strain delay effect of graphene starts to saturate at this concentration.

### Plastic response of NGCu and the strengthening mechanisms

**Figure 7:**
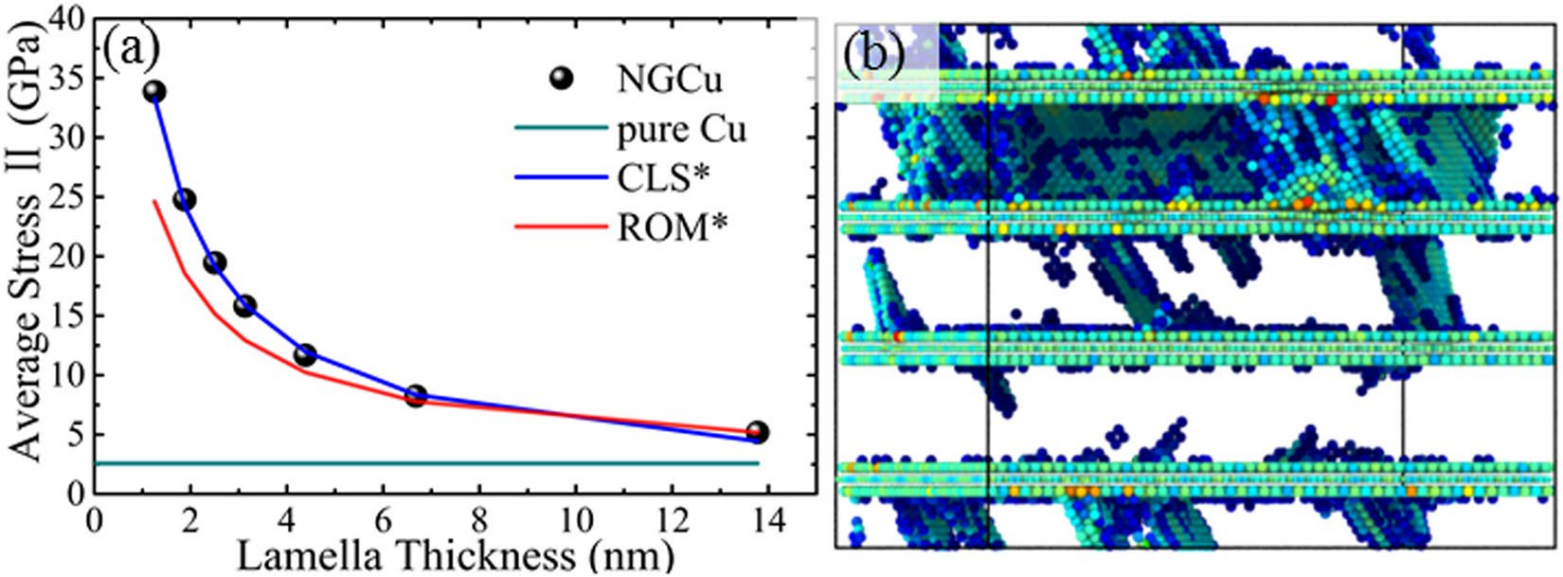
(**a**) The variation of average stress and (**b**) the atomic configuration of NGCu_4_ during plastic strengthening regime II.

As mentioned above, a rise of stress in regime II is caused by incorporating graphene monolayers. We average the stress in regime II to represent the strength of the NGCu and try to fit the ROM* model with the calculated average strength. As shown in Fig. 7(a), The ROM* model cannot agree well with the calculated average stress in regime II. With the λ decreasing, the average stress gradually exceeds the stress obtained by ROM* which means that another strengthening mechanism exist during this regime except for the load bearing effect contributed by the strong graphene layers. Figure 7(b) shows the atomic configurations of NGCu_4_ during this regime. It is shown that graphene/Cu interface acts as both the source and barrier of dislocation activity. Dislocations can hardly cross the graphene into adjacent layer due to the high strength of graphene. So the barrier effect of graphene/Cu interface leads to a strengthening in regime II compared with the pure Cu model until the curve reaching the point *β*. After point *β*, graphene layers are teared into fragments by the propagation of cracks induced by dislocation motion as discussed in Section 3.2. The CLS model is widely adopted to depict the strength of multilayer films where gliding dislocations are confined by the phase interface or the twin boundary^28,32,58^. Three terms are incorporated in the model to predict the strength increase with decreasing layer thickness, which respectively represent the stress propagating a glide dislocation loop, the contributions of the interfacial stress arose from the elastic deformation of the interfacial region and the interfacial dislocation array on the confined layer slip stress. The effect of interfacial stress may assist or work against the applied stress to cause yielding. To be specific, it would oppose the applied stress under tensile loading^59^, while for compressive loading, the interface stress would assist the applied stress. At the few to a few tens of nanometers length scales, confined layer slip of single dislocations is regarded as the effective mechanism, so CLS model is available in this case. Once dislocations can be easily transmitted across the interface and thus, CLS model become invalid to predict strength.

The GAZ model is also integrated into the CLS relationship as follows:

1 $${\sigma }_{CLS}=\frac{M\mu \ast b}{8\pi {h}^{{\rm{^{\prime} }}}}(\frac{4-\upsilon }{1-\upsilon }){\rm{l}}{\rm{n}}\,\frac{\alpha {h}^{{\rm{^{\prime} }}}}{b}-\frac{f}{h}+\frac{\mu \ast b}{L(1-\upsilon )}$$

where *M* is the Taylor factor, *b* is the magnitude of the Burgers vector of Cu; *h′* is the layer thickness parallel to the glide plane *h’* = *h*_*Cu*_/sin *θ*, *θ* is the angle between the slip plane and the interface; *υ* represents the Poisson ratio of Cu; μ* = *μ*_Cu·_*μ*_gra_/(*v*_Cu·_*μ*_Cu_ + *v*_gra·_*μ*_gra_) is the mean shear modulus of NGCu model which can be estimated by the shear modulus of each phases *μ*_Cu_, *μ*_gra_ and their volume fraction *v*_Cu_, *v*_gra_ using a relationship of ROM^19^; *α* represents the core cut-off parameter, *f* is the characteristic interface stress of multilayer, *L* is the mean shear spacing of glide array *L* = *bm*/*εv*_GAZ_, m is the strain resolution factor. The above revised CLS model is denoted as CLS*. With the parameters *M* = 3, *b* = 0.2556 nm, *θ* = 71.2°, *υ* = 0.343, *μ*_Cu_ = 48.3, *μ*_gra_ = 280, *α* = 0.5, *f* = 2 J/m^2^, *ε* = 0.2, *m* = 0.5^32,58,60^, the result calculated by CLS* is plotted in Fig. 7(a). The calculations fit well with the average stress data in plastic strengthening regime II, which further verify the application of the GAZ and the dimension thickness *h*_*GAZ*_. Therefore, it can be concluded that both the load bearing effect of graphene and the barrier effect of graphene/Cu interface synergistic strengthen the nanolaminated composites in the regime II.

**Figure 8:**
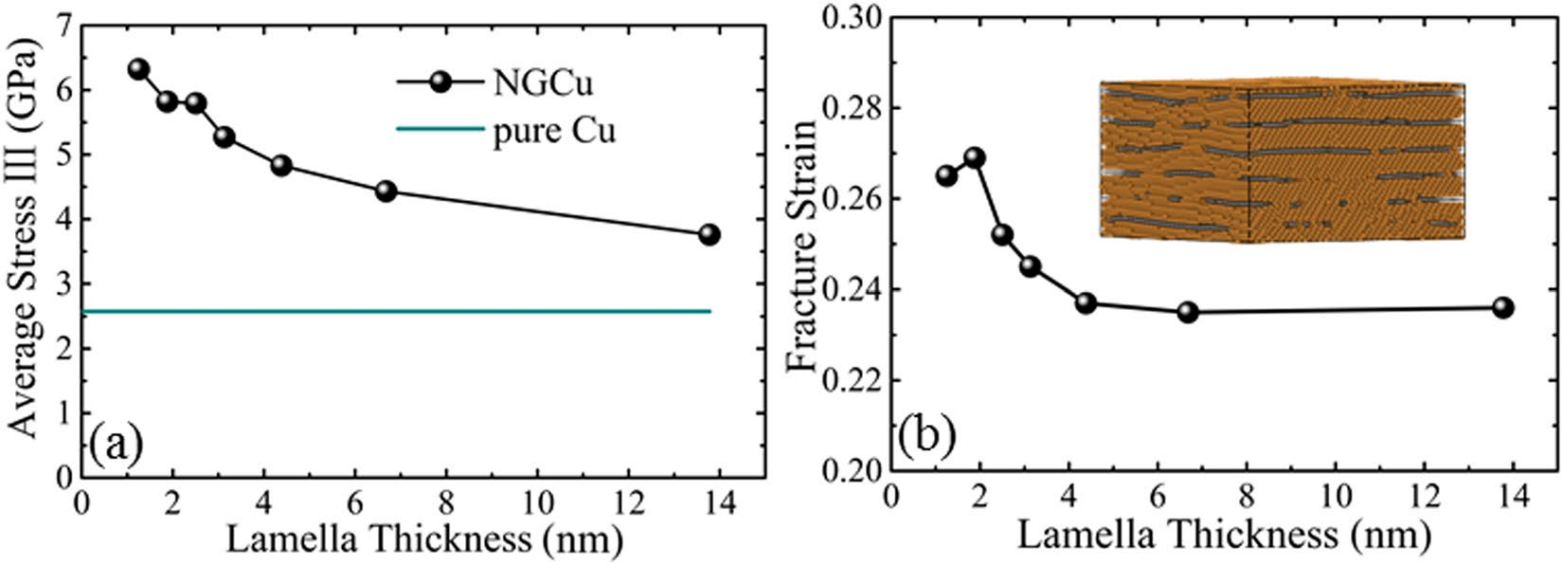
Variations of (**a**) the average stress in plastic flow regime III, (**b**) the fracture strain with various Cu lamella thicknesses.

After point *β*, the cracks propagate in graphene layers and the stress goes to a plastic flow plateau in plastic flow regime III. The average stress in this regime is calculated and plotted in Fig. 8(a). It can be found that the average flow stress rises with the *λ* decreasing. The strengthening effect of graphene monolayer still exists even though the graphene has ruptured. The fragments of graphene can still work as barrier to the dislocation motion, despite that the barrier effect is much weaker than that of the intact monolayer. Figure 8(b) shows the initial fracture strain of each NGCu model. The fracture strain increases with the decrease of λ until λ reaching 1.9 nm. After that, the strain decreases. Additionally, the rising tendency is more apparent with λ declining from 4 nm to 2 nm. Tracing back to the strain at the first peak point *α* where dislocation nucleates, the nucleation strain goes to a plateau when λ further declines from 1.9 nm. It is concluded that the strain delay effect of graphene does not accumulate with greater fraction of graphene with λ beneath 1.9 nm. A critical λ of 1.9 nm is thus obtained to reach both the best strength and ductility of NGCu material.

## Conclusion

In conclusion, we performed the uniaxial compression of nanolaminated NGCu with various Cu lamella thicknesses to investigate the effect of incorporated graphene and the underlying strengthening mechanism using molecular dynamics simulations. The main conclusions are summarized as follows:

1. The stress-strain curves of NGCu go through 3 regimes: the elastic regime I, and plastic regime II, III, in which different strengthening mechanisms work in corresponding regime. The transition of different mechanisms is related to their deformation behavior in different regimes.
2. It is shown that incorporating graphene monolayer can simultaneously contribute to the strength and ductility of NGCu composites. A critical λ of 1.9 nm is confirmed for the first time to effectively delay both the dislocation nucleation and fracture of NGCu composites in terms of corresponding strain, leading to effective improvement of the strength.
3. A GAZ model is established to account for the influence between the graphene and adjacent Cu layer. The revised ROM^*^ and CLS^*^ model integrated with GAZ are found to well describe the elastic properties of NGCu and the strengthening effect of incorporated graphene.
4. The fracture of graphene which leads to the failure of NGCu composites is caused by the approach of two Shockley partial dislocation lines lying above and beneath the graphene layer. The C-C bonds break along zigzag directions in graphene with a symmetrically distributed shear stress σ_xy_.
